# Spontaneous remission of fully symptomatic visceral leishmaniasis

**DOI:** 10.1186/s12879-015-1191-6

**Published:** 2015-10-23

**Authors:** Oussama Mouri, Mathilde Benhamou, Gaëlle Leroux, Nathalie Chartrel, Alain Devidas, Marc Thellier, Zahir Amoura, Nathalie Costedoat-Chalumeau, Pierre Buffet

**Affiliations:** AP-HP, Hôpital Pitié-Salpêtrière, Service de Parasitologie, F-75013 Paris, France; AP-HP, Hôpital Pitié-Salpêtrière, Service de Médecine Interne, F-75013 Paris, France; AP-HP, Hôpital Cochin, Service de Médecine Interne. Centre de référence maladies auto-immunes et systémiques rares, Service de Médecine Interne, F-75014 Paris, France; Hopital Sud Francilien, Service d’Hématologie, Corbeil-Essonnes, France; AP-HP, Hôpital Cochin, Service de Rééducation et Réadaptation, F-75014 Paris, France; UPMC Université Paris 06, Centre Immunologie et Maladies Infectieuses, F-75005 Paris, France

**Keywords:** Visceral leishmaniasis, Spontaneous remission, Hemophagocytic lymphohistiocytosis, Real time PCR, Giemsa stained, Bone marrow smear

## Abstract

**Background:**

Visceral leishmaniasis (VL), i.e., infection with *Leishmania sp.* associated with high fever, weight loss, massive splenomegaly and markedly altered laboratory parameters, is generally fatal if untreated. The possibility of transient spontaneous remission of fully symptomatic visceral leishmaniasis (VL) has been mentioned but, to our knowledge) has never been documented.

**Case presentation:**

We report the first documented history of a patient with overt, confirmed VL experiencing a complete remission in the absence of any anti-leishmanial therapy. The diagnosis of VL at the time of the self-resolving episode was strongly suspected based on clinical presentation and presence of antileishmanial antibody, then unequivocally confirmed years later by the presence of an amastigote on a stored smear and the positive quantitative PCR with *Leishmania*-specific primers from the material scraped from this same slide

**Conclusion:**

This report demonstrates that complete spontaneous remission may occur in patients with overt, fully symptomatic VL. VL should therefore be considered in cases of self-resolving or relapsing episodes of fever of unknown origin. Confirmation should be based on both serological tests and specific PCR on a blood sample.

## Background

Overt visceral leishmaniasis, i.e., infection with *Leishmania sp.* associated with high fever, weight loss, massive splenomegaly and markedly altered laboratory parameters, is generally considered fatal if untreated [1, 2]. The possibility of transient spontaneous remission has been mentioned [3] but, to our knowledge, has never been precisely documented because patients with documented VL are treated. We provide the first description of a complete, prolonged remission of fully symptomatic VL in a patient who had not received any anti-leishmanial therapy.

## Case presentation

In July 2008, a 27-year-old immunocompetent male patient was referred to our hospital for a 3-week history of fever, asthenia and pancytopenia. The patient had spent 4 month in an area endemic for *Leishmania infantum* in the South of France (Oriental Pyrennees). His medical history included dental infections and an allergy to methicillin. He reported two similar episodes of high fever and asthenia in 2006 (Table 1). The first episode in June 2006 had resolved spontaneously. During the second episode, in November, the patient was hospitalized with splenomegaly (17 cm), fever (40 °C) and weight loss (10 kg), anemia, leucopenia and thrombocytopenia (Table 1). Microscopic examination of a bone marrow (BM) aspirate was negative for parasites*.* The bone marrow smear showed normal cellularity and there was no indication of immunosuppression. All microbiological and immunological tests were negative (blood and urine culture, testing for HIV, salmonella, brucellosis, Lyme borreliosis, hepatitis A, cytomegalovirus infection, arboviral infection, and malaria) except for positive titers of anti-*Leishmania* antibody (IFA 1/400) and a positive serology for EBV suggestive of prior infection. In this context of fever of unknown origin, a presumptive 8-day course of intravenous antibiotic therapy (ceftriaxone and ofloxacin) was administered. All signs and symptoms, including fever, splenomegaly and asthenia, resolved in 4 weeks. No specific anti-leishmanial drug had been administered, as the treating physician had not retained this diagnosis*.* All laboratory parameters normalized during the same period, confirming complete remission of the episode (Table 1).

**Table 1:** ᅟ

	2006 Nov. 13th	2007, Feb. 1st	2008, July 2nd	2008, July 11th	2008, July 21st	2009, Jan. 15th
Antileishmanial agent				L-AmB Day1	L-AmB Day10	
Antimicrobial agents	Ciprofloxacin ceftriaxone					
Clinical parameters						
Temperature (°C)	40	37	40	38.2	36.1	36.5
Weight (kg)	59	73	68			72
Biological parameters						
White blood cell count (/mL)	1.480	7.000	1.280	0.990	4.110	9.200
Neutrophils (/mL)	0.720	4.018	0.563	0.495	1.993	6.522
Lymphocytes (/mL)	0.650	2.065	0.640	0.455	1.780	1.536
Hemoglobin (g/dL)	9.6	14.8	9.9	9.1	11.6	16.2
Platelet count (/mL)	87.000	197.000	75.000	64.000	250.000	182.000
ASAT (IU/L)	126	21	51	93		20
ALAT (IU/L)	56	18	15	19		17
C-reactive protein (mg/L)	160	1.9	63	97	4	
Ferritin (μg/L)	19.295	57	2.093			
Fibrinogen (g/l)	2.5	3.5	3.5			
Triglycerides (mmol/L)	2.32		2.21			
LDH (IU/L)	1.400	250	1.010			

In 2008, relapse occurred with weight loss (4 kg), fever (40 °C) and arthralgia (Table 1). Physical examination showed an enlarged spleen, measuring 20 cm on the subsequent CT scan. Antinuclear antibodies were positive (dilution of 1/1280) with a nonspecific pattern. The bone marrow (BM) aspirate showed hemophagocytosis. All microbiological tests, including *Histoplasma capsulatum* antibody were negative except anti-leishmanial antibody titers that were again positive (ELISA >1.7 for a threshold at 1)*.* Quantitative polymerase chain reaction (PCR) on a *L. infantum* kinetoplast DNA target found 30 parasites per mL, i.e., above the threshold for active VL (1 parasite/ml) [4]. Blood and BM cultures for *L. infantum* were negative.

Fever lasted 4 weeks before the diagnosis of VL was confirmed. The patient was then treated with liposomal Amphotericin B (l-AmB) (3 mg/kg/day D1-5 and D10) [5]. Complete fever resolution was obtained in 3 days along with a dramatic improvement of the patient’s general well-being. Ultrasonography showed regression of splenomegaly from 20 to 14 cm at the end of the first week, followed by normalization to 12 cm 6 months later. At that time laboratory parameters had returned to normal levels (Table 1) and quantitative PCR for *L. infantum* was negative. When last seen, in September 2010, the patient was completely asymptomatic.

**Fig 1:**
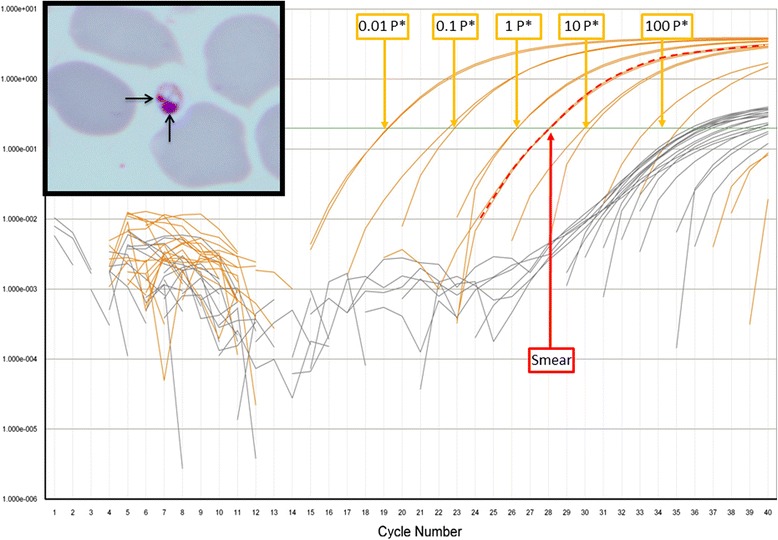
Amplification profile in RT-PCR of the Giemsa slide of the bone marrow smear, the negative blood slide and the negative tissue slide, the amplification of the *Leishmania* kinetoplast DNA is labeled in orange and the amplification of the DNA of the Internal Positive Control (IPC) is labeled in Grey. The amplification of the kinetoplast DNA of *Leishmania* from the Giemsa slide of the bone marrow smear is indicated by red vertical dotted line and occurred at 28 CTs which corresponds to the presence of 1.8 parasites on the slide. The amplification of the kinetoplast DNA of *Leishmania* from the five positive points of the range are indicated by blue vertical dotted lines. Typical amastigote form of *Leishmania sp* (2–3 mm in diameter) seen in smear bone marrow shown in insert squares at the left side of the figure. The nucleus and kinetoplast are in red arrow. Staining May Grunwald Giemsa (MGG) x1000. *Parasite

We reexamined a bone marrow smear performed in 2006 during the first hospitalization. Independent, complete examination of this smear by 2 observers (10 h of observation under the microscope) revealed the presence of a single typical amastigote form of *Leishmania* sp. (Fig. 1). Material on the smear was then thoroughly scarped from the slide with a blade and resuspended in 30 μl of sterile distilled water. Real time PCR amplification was performed in a thermal cycler TAQMAN® (Applied Biosystems) using Leishmania kDNA specific primers as described by Mary et al. [4]. One negative blood slide and one negative tissue slide were used as controls. Amplification of the material retrieved from the slide occurred at 28 CTs, corresponding to the presence of 1.8 parasites (Fig. 1).

## Discussion

This is the first documented report of a patient with overt, confirmed VL experiencing a complete remission in the absence of any anti-leishmanial therapy. The diagnosis of VL at the time of the self-resolving episode was strongly suspected based on clinical presentation and presence of antileishmanial antibody, then unequivocally confirmed years later by the presence of an amastigote on a stored smear. Quantitative PCR from the material scraped from this same slide using *Leishmania*-specific primers was positive. The excellent specificity of this PCR has been confirmed by several teams [4, 6–9]. The last episode, during which PCR was positive in a blood sample was followed by a positive outcome under anti-leishmanial therapy with liposomal amphotericin B. No relapse occurred during the 2 years following administration of anti-leishmanial therapy, which is consistent with the high efficacy of l-AmB in Mediterranean VL [10].

It has been known for decades that patients with mild symptoms of VL may recover spontaneously and that a minority eventually develop overt clinical VL [11]. In *L. infantum* transmission foci, asymptomatic infections and mild clinical forms of VL are indeed frequent in humans [12, 13]. This is different from the spontaneous remission of fully symptomatic severe VL, as described here. Uncontrolled studies that show improvement or apparent cure in patients receiving experimental anti-leishmanial interventions for overt VL should thus be interpreted cautiously [14, 15]. We cannot definitely exclude the hypothesis that the short course of intravenous antibiotic therapy (ceftriaxone and ofloxacin during 8 days) during the first episode may have contributed to the patient's improvement. A few reports in vitro or in animals suggest indeed that quinolones may have some activity against *Leishmania* [16–18] thus possibly contributing to the control of parasite loads. Thereafter, the patient was appropriately treated and cured with liposomal Amphotericin B.

For confirmation of VL, PCR in the blood is more sensitive than conventional search of the parasite even in bone marrow aspirates. Time spent for reading Giemsa-stained smears in the search for *Leishmania* amastigotes increases the test sensitivity that raises to 95.4 % after 60 min and 89.7 % when 1.200 fields are examined [19]. When a macrophage activation syndrome is present, repetition of bone marrow aspirates also increases diagnostic sensitivity [20]. Why patients would spontaneously recover from overt VL is not clear. Interestingly, our patient met five out of the eight criteria of the hemophagocytic lymphohistiocytosis (HLH-2004 revised diagnostic guidelines) [21]. Therefore, if HLH had also occurred during the initial episode, phagocytosis of *Leishmania*-infected cells by previously activated macrophages, or a general activation of macrophages as observed in HLH, may have contributed to the control of parasite loads. This hypothesis is consistent with the absence of *Leishmania* amastigotes in bone marrow aspirates in 36.3 % of patients with VL and HLH [22].

## Conclusion

In summary, this report demonstrates that complete spontaneous remission may occur in patients with overt, fully symptomatic VL. VL should therefore be considered in cases of self-resolving or relapsing episodes of fever of unknown origin. Confirmation should be based on both serological tests and specific PCR on a blood sample.

## Consent

Patient was informed of the process by this attending physician using a procedure common to all French National Reference Centers (NRC) (http://www.parasitologie.univ-montp1.fr/conseil.htm) and gave his oral consent for data collection and publication. Mention of this consent was written in the medical chart.

## Abbreviations

VL: Visceral leishmaniasis
PCR: Polymerase chain reaction
HIV: Human immunodeficiency virus
EBV: Epstein-barr virus
HLH: Hemophagocytic lymphohistiocytosis
